# First Annual Report of the Registrar-General of Births, Deaths, and Marriages in England

**Published:** 1840-04

**Authors:** 


### Art. II.

*First Annual Report of the Registrar-General of Births, Deaths, and Marriages in England.—London,* 1839. 8vo, pp. 168.

Before presenting our readers with a view of the contents of this very important volume, we think it may be useful to offer—not an apology for statistics, for this science stands in need of no apology—but a vindication of it from certain imputations to which we have observed it subject. The first we shall notice is, that the study is a cold-hearted one—that it leads its votaries to regard their fellow-creatures as mere abstractions, or even as "ciphers," and not as human beings, with the thoughts, feelings, and aspirations of men within them. Now, we feel convinced that it would be impossible for any one to pursue statistics in such a spirit; if he saw the "ciphers" only, they would be speedily abandoned, as the most wearisome of objects. It is the suggested ideas—all which ideas refer to man in the concrete, to man as a being possessing happiness to be augmented and misery to be relieved—which invest this science with all the interest that appertains to it. The figures of a statistical table suggest various things to varying minds: the statesman sees in them one thing, and the physician another; and even in the two classes to which these belong, the ideas will vary according to the benevolence, talent, and previous information of him who contemplates the "ciphers;" but the suggested ideas will ever have a relation to the improvement of the moral and physical condition of man. However strong the objections to statistics in general, those against the application of the science to the improvements of practical medicine have been still more vehemently urged. The impossibility of applying the precision of numbers to phenomena so fleeting and variable as those of disease has been the theme of many a pen. Now it would be no difficult thing to show that numbers lie at the root of almost all our opinions in practical medicine; but these numbers, however convincing to the mind by which they are estimated, are not communicated to others in a form which wins for them confidence, or constitutes them safe grounds of reasoning. An example or two will best illustrate this position. The first we shall take from the excellent letter of Mr. Farr to the Registrar-General, which forms so important a part of the Report before us:

"The prevalence of a disease, for instance, is expressed by the deaths in a given time out of a given number living with as much accuracy as the temperature is indicated by a thermometer; so that when the mean population of the district is known, the rise and decline of epidemics may be traced exactly, and it will then be possible to solve the problem, whether certain tribes of epidemic disorders constantly follow others in one determined series or cycle Loose phrases are still current, for which numerical formulæ will be substituted.—Sydenham, one of the most accurate of medical writers, in speaking of smallpox, employed such terms as these: (1661) 'It prevailed a little, but disappeared again.' (1667-9) 'The smallpox was more prevalent in town for the first two years of this constitution than I ever remember it to have been.' (1670-2) 'The smallpox arose; yielded to the dysentery; returned,' &c. &c. These terms admit of no strict comparison with each other; for it is difficult to say in which year the smallpox was most fatal, and impossible to compare Sydenham's experience thus expressed with the experience of other writers in other places and other ages; for 'prevailed a little,' 'raged with violence,' and similar terms, may imply either that smallpox destroyed 1, or 2, or 5, or 10 per cent. of the population. The superior precision of numerical expressions is illustrated by a comparison of Sydenham's phrases with the London bills of mortality in the same years." (p. 87.)

Mr. Farr gives a table containing the mortality from smallpox during each year from 1661 to 1680 inclusive, and then remarks:

"The 1987 deaths from smallpox in 1668, and the 951 deaths from that disease in the year following, express the relative intensity of smallpox in distinct terms. The method of the parish clerks, although imperfectly carried out, was the best. Sydenham guessed the quantity, and called it great or small; the parish clerks measured it, and stated the result in figures." (p. 88.)

So with regard to the treatment of disease, an experienced physician pursues one line of practice, and avoids another, because he has found the one he adopts more generally successful. Here numbers are mentally compared, and when he is requested to count, he is merely asked to do in a precise form what he has constantly been doing in a vague one,—to reduce his experience to a state which admits of comparison with that of others, and renders it an augmentation of the sum of general knowledge, instead of one which is conclusive to his own mind only. Admitting that age, sex, temperament, and idiosyncracy modify diseases so much, that there is not often a perfect identity between individual cases, and that something special may be required in the conducting of many of them; yet a general accordance will be found in the treatment of diseases of the same kind by the same practitioner. Let us take rheumatism as an example: one man may prefer for its treatment blood-letting, another calomel and opium, another antimony, colchicum, &c.; but, however individuals may differ, each will be found to pursue a consistent plan in the treatment of this disease. If each, then, would throw an account of his successes and failures into one common stock, conclusions beneficial to therapeutics might be deduced; for, though the frequency of specialities in individual cases might, according to the well-known doctrine of probabilities, render such a group as any medical man could furnish from his own practice, an insecure ground of reasoning; yet as contributions to some extensive fund, in which, from its great amount, incidental irregularities should compensate each other, and which should furnish the means of comparing together very numerous cases of various diseases, grouped according to correct resemblances, the various groups being similarly and dissimilarly treated, all facts faithfully recorded and well arranged, would be of great value. What appears to be wanting is the machinery for applying the numerical method to therapeutics, on a scale sufficiently ample to obviate the errors which might arise from special and exceptional cases abounding in small groups. Such a Society as the *Société Médicale d'Observation*—of which we have already given some account, and of which, whilst we have censured its abuse of the numerical method, we have acknowledged the plan to be admirable* —established in this country, and receiving statistical reports from its various parts, would meet, as it appears to us, the object to be attained, *if it be attainable by means of statistics.* The main argument against such an expectation, that "the observation necessary for the safe treatment of diseases comprehends many circumstances which distinguish *every* individual case from the rest," we think too broadly stated. *Individual* diseases (cases of disease) resemble each other; they admit of arrangement founded on such resemblance; and, according to the degree of it, are classified into species, genera, orders, and classes. Now it does appear somewhat extraordinary, that their safe treatment should never depend upon the circumstances in which they agree, but uniformly upon those by which they are distinguished. This appears contrary to the whole analogy of nature, and, what is still more important, we venture to assert will be contradicted by the practice of almost every physician in the world. He will be found treating his cases of pneumonia, &c., by means the same at least in *kind,* though in *degree* they will be varied according to the age, strength, and sex of the patient. The contrary supposition, that his practice was founded on the distinctions, not on the resemblances, would be imagining every practitioner to be a law to himself, and would amount to the total abrogation of what little therapeutic principles we possess. If diseases themselves admit of classification, which, as is justly observed in the Registrar-General's Report, "is another name for generalization," and if "successive generalizations constitute the laws of the natural sciences;" if, too, it appears, that this kind of statistics is rapidly teaching us that the circumstances which induce diseases, and the correction of which prevents them, admit of classification—it seems incredible that those circumstances which modify their progress beneficially, in one word, their treatment, should be utterly abnormous. The argument which has sometimes been advanced against the application of numbers to therapeutics,—and which we ourselves have used,—that a man must first consult his tables, and make his calculations, before he can act in any given case, is not to be admitted without much qualification; as thus broadly stated it involves a misconception of the mode in which statistics (if it can improve) must improve therapeutics. The long-continued and conjoint labours of many must first furnish an ample store of well-observed and well-arranged facts, and from these general principles must be deduced, and become the common stock of the profession. We are by no means certain that this can be done, but we are certainly of opinion that it ought to be attempted, and we very much misconceive the character of the members of our profession, if they are not found zealous collaborators in such a work, should it once be instituted.

These hopes of benefit to be conferred upon the most important branch of the healing art by statistics, have not been engendered by the Regis-

* See Vol. V., p. 154, of this Journal.

trar-General's Report: for in the early part of our career we expressed such hopes;* but they have certainly derived additional vigour from the mass of precise information on the causes, prevalence, and diffusion of disease which this Report imparts. We shall now proceed to convey to our readers such portions of this information as we deem most material, and our limits will admit, premising that the toil of analyzing tables and arranging the results of such analysis has been much abridged by the valuable letter, including tables and abstracts of the causes of death, by Mr. Farr, which forms so important a part of the Report.

In framing the general abstracts of the number of marriages and births, the Registrar-General has not attempted to specify the localities, but has given one statement for the whole kingdom of England and Wales, only distinguishing the number of marriages or of births registered in each quarter of a year. In the abstract of deaths, however, he has entered into more minute details, and has assigned the following very satisfactory reasons for his proceeding:

"Such details are of acknowledged value, as data for determining the laws of mortality—as bases for calculations affecting the interests of millions. Tables exhibiting the proportion of deaths at every successive year of age are among the most important materials from which are deduced the true principles on which should be founded the systems of life annuities and of life insurances, and the rules of friendly societies established for the use of the poorer classes. The materials hitherto accessible are admitted to have been too limited for framing, satisfactorily, tables to regulate the amount of contribution at various ages, by which members of such soeieties may become entitled to allowances in old age, or to sums payable at death. The insufficiency of the data hitherto collected, and the contradictory nature of the several tables founded upon them, are strongly set forth in the Report of the Select Committee of the House of Commons, in 1827, on the laws respecting friendly societies. It is there stated that, 'according to the Northampton Tables, out of 1000 persons existing at the age of twenty-five, there survive at the age of sixty-five 343 persons. By the Carlisle tables, no fewer than 513 persons will survive;' whereby it appears, 'that a society which should adopt the Northampton Tables would, if the mortality among its members should correspond with the Carlisle Tables, have *three* annuitants where it calculated upon *two*. Of those annuitants, moreover, a larger proportion would live to enjoy the annuity for a considerable number of years; for instance, of the 343 persons who would be annuitants according to the Northampton tables, ninety-eight would live for fifteen years; according to the Carlisle Tables, 162 persons would survive through that period, and attain the age of eighty years." (pp. 15-16.)

Still further, to show the necessity for better ascertained facts to elucidate these important subjects, the Registrar-General annexes, in a tabular form, the results of seven approved tables of mortality, the discrepancies among which are excessive. The first two columns will suffice to render these manifest. Of 100,000 persons aged twenty-five, there would be alive at the age of sixty-five, according to Dr. Price's table, founded on the births and burials at Northampton, 34,286; the first Swedish tables, as published by Dr. Price, for both sexes, 43,137; M. De Parcieux's tables, founded on the mortality in the French tontines prior to 1745, 51,053; Mr. Milne's table, founded on the mortality observed at Carlisle, 51,335; Mr. Griffith Davies's table, framed from

* See the review of the work of M. Louis, "*Sur la Saignée*," &c., in our Second Number.

the experience of the Equitable Life Insurance Office, 49,330; whilst of Mr. Finlayson's tables, founded on the experience of the Government Life Annuitants, the one, as mentioned in his evidence in 1825, makes the survivors 53,470; and the other, stated in his evidence in 1827, rates them at 53,950. Of 100,000 persons aged sixty-five, there would be alive at the age of eighty, according to the same authorities respectively, 28,738, 23,704, 29,873, 31,577, 37,267, 38,655, 37,355.

These discrepancies are certainly sufficient to justify the recommendation of the Report of the Committee of the House of Commons, that measures be adopted for making "an accurate and extensive collection of facts," whereby may be facilitated "the solution of all questions depending upon the duration of human life." Mr. Lister prudently abstains from making an estimate of the probable duration of life at successive ages from the abstracts of deaths for a single year; but confines himself to recorded facts, accompanied with deductions that are clear and unimpeachable, justly remarking that each year's accumulation will increase the value of the records, by augmenting the number of facts on which calculation may be brought to bear.

Not only were there great discrepancies in the estimates of mortality as regards the general population, but there was an entire want of all data by which districts widely differing as to the occupations of the inhabitants, and other circumstances by which their health and longevity would be especially influenced, could be compared, or rather discriminated. It was justly stated by Mr. Milne to the Committee on laws respecting "Friendly Societies," that "no one scale of contributions could, with propriety, be adopted by all such societies; but that one composed of members living in or near a manufacturing town required a table very different from that which would be required in places where the population was less dense, and where a considerable proportion of the members were chiefly employed in the open air; but that these were differences which he could not presume to estimate, for want of *data*." The actuary of the National Debt Office gave testimony of the same purport to the Committee on Parochial Registration in 1833.

The Registrar-General, mindful of the various circumstances influencing public health and longevity, has not confined himself to an abstract of deaths for the whole kingdom of England and Wales, but has felt how important it was, to use his own language, "to compare town with country—agricultural districts with manufacturing and mining districts—the hilly with the low and level—the maritime with the inland—the eastern and northern with the western and southern parts." In carrying out this plan, he remarks, "I have had regard not so much to the observance of established boundaries as to those circumstances from which diversity may be expected to arise; and I have in some instances included in the same table contiguous counties similar in soil, climate, elevation, and the employments of the people; and have, in other instances, disregarded the boundary of the county, where it was desirable to compare two large portions of its inhabitants pursuing very different occupations. The expediency of causing each division to consist of integral unions, or superintendent-registrar's districts, has also precluded a very strict adherence to the boundaries of the counties, there being many unions situated on the outward verge which include portions of two or more counties." (p. 18.)

The amount of registration during the first year is—births 399,712, deaths 335,956, marriages 111,481. The Registrar-General thinks that, with respect to the registration of deaths, the system has, during the first year, been eminently successful. The registration of births has been less perfect than that of deaths; and we regret to observe that the Registrar-General ascribes this comparative imperfection, in a considerable degree, to an extensive and stubborn opposition. It is consolatory, however, to observe, from the increase of the entries during the third and fourth quarters, that the success of this branch of the plan is progressive, and that the various impediments opposed to it are not continuing to prevail against it.

The whole of England and Wales is divided into twenty-five districts or divisions, according to the principle formerly mentioned; the statement of the population in each district being taken from the census of 1831, the latest source of information on the subject given on authority. This statement is, of course, considerably below the reality; although a close approximation might be made to the present population of each district, the population of 1831, being common to all, answers perfectly well the purpose, when the object is to compare the relative salubrity of districts. Besides the population, the number of families, according to the same census, in each, and the proportion engaged in agriculture, trade, manufactures, and handicraft, and of families whose occupations are not referrible to either of these heads, and the area of each district in acres, are stated. The whole plan appears extremely well devised for the discovery of certain general sources of health and longevity, and their opposites; for showing how far these conditions are influenced by the concentration of the people in towns, their diffusion over a wide surface, their occupation in agriculture, manufactures, or mining, and by the nature of the surface, whether mountainous or plain; and, by a little further scrutiny into the food and other circumstances of the inhabitants of the respective districts, for the illustration of most subjects of interest to the statistical enquirer.

Examples of the influence of certain of the circumstances mentioned over the duration of life are readily deducible from the tables. In the whole of England and Wales, out of 1000 deaths, 145 have been at the age of seventy and upwards; while in the thinly-peopled districts of the north riding and the northern parts of the west riding of Yorkshire, and in Durham, except the mining districts, the proportion has been as high as 210. In Northumberland (excluding the mining district), Cumberland, Westmoreland, and the north of Lancashire, the proportion has been 198; in Norfolk and Suffolk, 196; in Devonshire, 192; and in Cornwall, 188. Where the population is densely congregated, the results are very different. In the metropolis and its suburbs, the proportion who have died at seventy and upwards has been only 104; in Birmingham, 81; in Leeds, 79; and in Liverpool and Manchester, only 63. In the smallness of the proportion of such deaths in the four large manufacturing towns, as compared even with the metropolis, we trace the influence of employments in addition to that of concentration. The operation of both the causes of mortality just mentioned seem to be rendered manifest by a comparison of the reports from Division 22, including the north riding of the county of York, with such portion of the west riding as is not comprised in Division 20, and the county of Durham (*except the mining parts*), and that of Division 23, comprising the *mining parts* of Northumberland and Durham, and the populous towns of Newcastle, Sunderland, and South Shields. Division 22 has a population of 319,042 on an area of 2,104,736 acres; Division 23, has one of 318,941, or within a trifle of the same amount, on an area of 688,708 acres, or less than one third of that of Division 22. Of the 66,175 families contained in the 22d Division, 25,082 are engaged chiefly in agriculture; and of the 69,532 families contained in Division 23, but 7,298 are so occupied. In the former of these divisions, we find the mortality to be, within the year, 6,953, or one in forty-five of the population, according to the census of 1831; that of the latter 8,112, or one in 39·3 of the population, according to the same estimate. Of the 6,953 deaths in the former division, 1,363, or nearly one fifth, took place at the age of seventy or upwards; whilst in the latter, 1,101, or about one eighth of 8,112, occurred at the same period of life. The mining parts of Staffordshire and Shropshire, compared with the adjacent rural district, present a similar deficiency of deaths in old age, and an excess of general mortality, so far as that can be deduced from the population of 1831, such as have been pointed out in the case of Durham and Northumberland.

The Registrar-General observes a very marked diversity in the proportion of deaths of infants in different parts of the country. In the mining parts of Staffordshire and Shropshire, in Leeds and its suburbs, and in Cambridgeshire, Huntingdonshire, and the lowland parts of Lincolnshire, the deaths of infants under one year have been more than 270 out of 1000 deaths at all ages; while in the northern counties of England, in Wiltshire, Dorsetshire, and Devonshire, in Herefordshire, Monmouthshire, and Wales, the deaths at that age, out of 1000 at all ages, scarcely exceeded 180.

Some supposable sources of error are pointed out to those who might be disposed to draw conclusions from the tables. In the 1st (metropolitan) Division the deaths of persons between the ages of twenty and fifty are 241 out of 1000 at all ages; whilst in Division 6, comprising the counties adjacent to the metropolis, they are only 192; but it is discovered, from the enumeration made in 1821, that of 10,000 persons living in Middlesex, there were 4,522 between the ages of twenty and fifty, and in Bedfordshire, Buckinghamshire, and Hertfordshire, but 3,581 persons of the same age; a difference attributable to persons born in the counties near London quitting them, as they emerge from childhood, to seek employment in the capital. A similar explanation is found of the great discrepancy in the proportion of deaths under five years of age in Division 19, comprising Lancashire, south of Morecombe Bay, with the exception of Manchester and Liverpool, and the same in Division 25, comprising Herefordshire, Monmouthshire, and Wales. In the former division, according to the enumeration of 1821, there were, out of 10,000 persons, 3,293 at the period of life mentioned; in the latter, 2,754. As warnings and corrections to erroneous calculations, Mr. Lister has appended to the abstract of deaths the table containing the ages of persons in the several counties of England, on the 28th of May, 1821, with the addition of the same for Wales.

We now turn to those portions of the Report which fall most immediately into our own province—the letter of Mr. Farr, and the tables containing an abstract of the causes of death in the twenty-five divisions. We shall have ample occasion to express our conviction that justice has been done to this very important subject by Mr. Farr; but no pains taken by him can give the Report the full value of which it is susceptible. This must be derived from the correctness of the *items;* and here all depends upon the body of medical practitioners throughout the kingdom : and, with great confidence in their intelligence and disposition to render a public service, we yet would impress upon them the importance of the duty they are now called upon to discharge, without any reward but the conviction of a benefit bestowed upon their fellow-creatures. If their own ideas of the cause of death are accurate in any given case, it will be less trouble to assign the real than a fictitious one to the registrar, and consequently an inaccurate return will be an impeachment of the soundness of their own judgment: so that, besides the well-being of the community, and the advancement of knowledge (terms which are well nigh convertible), motives of powerful influence over a profession preeminent in science and benevolence, regard to individual character should secure the accuracy of the return. The omission or refusal to give information to the registrar, seeing that it is done with little or no trouble to the medical man, is a case we cannot contemplate, or, of course, discuss.

The first object which arrests our attention is the statistical nosology. Diseases are arranged under the heads of, 1st, epidemic, endemic, or contagious diseases, and 2d sporadic diseases, whilst deaths by violence constitute a 3d division in the nosology. Of the first class, the species are simply enumerated ; but those of the second are divided according to their seat in the nervous system, in the organs of respiration, of circulation, of digestion, in the urinary organs, in those of generation, of locomotion, in the integumentary system, and of uncertain seat. The diseases thus classified amount to nearly 200, and, as necessarily *only* fatal diseases are comprehended, the arrangement seems sufficiently ample without needless minuteness. The names recommended to be used are given in a first column, and a second column comprehends synonymes and provincial terms. Mr. Farr is evidently anxious that precise terms should be employed. He objects, for instance, to the term inflammation of the bowels, as not distinguishing peritonitis from enteritis; and contends, very properly, with M. Louis and, we believe, all good pathologists, for the limitation of the word *enteritis* to inflammation of the internal membranes of the bowels, whilst *peritonitis* designates inflammation of the peritoneum, whether lining the abdominal muscles or investing the intestines. In these views we perfectly accord.

Mr. Farr is very properly anxious that a uniform statistical nomenclature should be adopted, and indicates the prevailing sources of confusion, such as designating one disease by various terms, applying the same term to different diseases, employing vague terms, and registering complications instead of primary diseases. This latter point he exemplifies by pneumonia occurring after hooping-cough, and proving fatal; and he would, in this case, refer the death to the primary disease, and adhere generally to this principle in similar instances. It appears to us that the members of the profession should be universally informed of the nomenclature and plan of registration most suitable to the purposes of those with whom will rest the labour of arranging the innumerable items received from various quarters, and making the proper deduction from them; and this can be accomplished only by furnishing all medical men with a sketch of Mr. Farr's classification, and the pertinent remarks on nomenclature with which it is accompanied; and this might be accomplished, one would suppose, through the superintendent-registrars, at small expense of money and trouble.

In table A, at page 120 of the volume, will be found an abstract of the causes of death registered in England and Wales, from July 1st to December 31st, 1837, both inclusive. The number of deaths registered during this half year is 148,701, of which 75,159 occurred in males, and 73,542 in females. The population of the two countries is estimated according to the population of 1831, with an addition framed according to the decimal increase which has been calculated at four periods, and found to be very uniform. Corrections were required, in the first place, for a deficiency in the enumeration of males; and again, for the deaths which escaped registration during the first two or three days of the first quarter, and could not receive the compensation which would necessarily occur in each succeeding quarter, those failing to be registered in the second being compensated for from the first, those in the third from the second, and so successively. With these corrections, the annual mortality was found to be, males 2·08, females 1·97, and mean of the two sexes 2·02 per cent.; or, in other language, males 1 in 48, females 1 in 51, and mean of the two sexes 1 in 49. This estimate is formed from the latter half of the year 1837, during the early months of which influenza prevailed epidemically. The latter portion of this year was, as is well known to the profession, very healthy; a condition, be it remarked, very generally existing, for a considerable period after epidemics; we should, consequently, conclude, that the estimated mortality, though there is every reason to think very accurate for the period, is below the average mortality of the country for a series of years.

The same table furnishes much valuable information on the relative prevalence of various classes of diseases, and of individual diseases. The first class, comprising those which are epidemic, endemic, and contagious, was fatal to 32,537, of whom 16,190 were males, and 16,347 females, being in the proportion, relatively to the numbers of the two sexes, of 4·7 to 4·6 per 1000 annually, and of 4·651 per 1000 for the mean of the two sexes. Smallpox, croup, thrush, diarrhœa, dysentery, and cholera, proved more fatal to males than females; while influenza and hooping-cough, particularly the latter, cut off a greater number of females; and typhus, erysipelas, scarlatina, and measles affected both sexes equally. The mortality from smallpox, measles, scarlatina, hooping-cough, croup, thrush, and diarrhœa, constituting 3·036 per 1000 of the entire mortality from epidemic, endemic, and contagious diseases, occurred principally among children; although, it is stated, a considerable number of adults were carried off by these diseases. Cholera, dysentery, *influenza*, ague, typhus, erysipelas, and syphilis, constituting the remaining 1·615 per 1000, attacked adults chiefly, though they did not entirely spare children. The conclusions regarding certain of these latter diseases are deduced from a small number, but they are, nevertheless, consonant with the general experience of the profession. The total number of cases of influenza given in the table is 484; but the report of Mr. Farr comprises, it will be remembered, only the latter half of 1837; and it was during the first quarter of this year that influenza was so extensively epidemic. The results of this registration, however, confirm the recorded opinion of a numerous body of practitioners, formed from observation of the epidemic in its intensity, as may be observed on reference to the "Report on Influenza," contained in the 6th volume of the "Transactions of the Provincial Medical and Surgical Association."

Smallpox destroyed 5,811 in the half-year. This disease was epidemic in several parts of the country, particularly in Liverpool, Bath, and Exeter. Of 1,056 deaths which occurred from it in Bath, Exeter, parts of Shropshire, Worcestershire, and the metropolis, 887, or considerably above four fifths of the whole, took place in infants under five years of age; and Mr. Farr regards the statement of these ages as an approximation to the ages of the whole 5,811. He remarks, with truth, how far the fact mentioned would go to settle the question as to the progressive decrease of the preventive power of vaccination from the period of its performance, provided the medical attendant would ascertain whether the individual dying of smallpox had been vaccinated, and the interval between his being so and his death, which might easily be done. He thinks that vaccination is too long delayed by all classes, and quotes the early deaths at Bath and Liverpool, to prove that it should be performed within the third month. We find that, at the two places mentioned, twenty-two children died of smallpox between birth and the second month, forty-one between the third and fifth month, fifty-four between the sixth and eighth, and fifty-nine between the ninth and eleventh, making a total of 176 deaths within the first year of age. Of those in the second year, there perished 158; and in the third, 110. The numbers then steadily decrease till we reach the tenth year, and in that period of life only three died; and at all ages above ten, but thirty-five. We need scarcely remark how great a value such a statement as this would have possessed, had it been connected with the information relative to vaccination which the reporter desiderates. As it stands, it furnishes an argument, though not a positive one, in favour of vaccination; for we find a progressive decrease of mortality from smallpox in proportion as the probabilities of this operation having been performed increase.

Under sporadic diseases of the nervous system, 21,852 deaths (fifteen per cent. of the total number) were registered. The mean annual rate of mortality from the whole group was 3·1 per 1000; but males suffered more than females, in the proportion of 3·4 to 2·8. Paralysis, chorea, and epilepsy were the only diseases of this class which affected more females than males. Cephalitis, hydrocephalus, and convulsions, the diseases chiefly but not exclusively incidental to young children, cut off 2·4 males and 2·0 females out of 1000 living; apoplexy, ·42 males, ·35

females; tetanus, ·013 males, ·003 females; delirium tremens, ·025 males, ·002 females. Mr. Farr justly remarks, that tetanus generally follows wounds, and is therefore remotely caused by accidental violence, to which males are more exposed than females. He remarks, too, that delirium tremens is also sometimes brought on by wounds in drunkards, and in persons exhausted by passion or misery. Admitting that traumatic delirium is, in its outward manifestations, very much allied to delirium tremens, if not identical with it, in other classes besides those mentioned, we should yet demur to the opinion intended to be conveyed in this sentence, being convinced that females under similar circumstances of intemperance are very much less prone to this disease than males. We would observe, that the whole number of cases of this affection reported as having been fatal during the half year is so small, being only ninety-five, of which eighty-six were in males, nine in females, as to convince us that cases which ought to have been returned under this head have found their way into that of cephalitis or some other disease.

In the class of diseases of the nervous system, ten deaths, nine of females, one of a male, are ascribed to mental emotions of one kind or another; seven to fright, one to grief for the death of a son, and two to a broken heart. In certain of these, the alleged cause and result occurred within so short a period of each other, as to render the causation less equivocal than it frequently is in such cases. For instance, a female, aged sixty-three, died on November 7th, from trouble for the death of her son, which took place October 30th; and, again, the death of a female, aged forty-one, which occurred November 14th, is ascribed to "fright," occasioned by the sudden death of a brother, and her brother died on the 19th of October. Under the same head are noted several curious cases, such as laryngismus stridulus, one male, one female, aged 100, (we copy exactly, but, though the punctuation would lead to the conclusion that the parties were both of the same age, it appears probable that this is a typographical error, and that the female only was 100;) chorea, a female, aged seventy-five; hysteric fits, one male, aged sixty-seven, two females, aged seven and fourteen days, and others which we pass over, that we may notice the diseases of the respiratory system.

From this class there arose 38,522 deaths, or twenty-seven per cent. of the total number; of these, 27,754, or twenty per cent. of the total deaths, were owing to phthisis. The deaths from this dreadful disease in males amounted to 12,968, in females to 14,786, the ratio being 4·155 of the latter to 3·771 of the former. The mortality from all diseases of these organs was equal in males and females; bronchitis, pleurisy, pneumonia, and asthma destroying more males than females. The deaths from the whole class were in the proportion of 5·5, and of phthisis in that of 4 per 1000 of the population annually. These proportions correspond very considerably with the estimates of recent statists regarding the general population; but they are very much lower, indeed, than those deduced by Major Tulloch from the mortality of the troops in England, especially from that which occurs in the foot-guards. Mr. Farr's law of the mortality from this disease deserves to be recorded. He says, "it may be laid down as a general principle, that wherever the proportion of deaths from phthisis, compared with the total deaths, is high, the absolute mortality is low, and that the absolute mortality from phthisis itself is low. The deaths out of the living express the real tendency to phthisis."(p.116.) This sounds somewhat paradoxical, but converted from the abstract to the concrete, it will be found to be true. In Great Britain, the deaths from phthisis constitute one fifth of the total deaths—a high proportion; but the total deaths are but twenty annually out of 1000 living—a low mortality; and the deaths from phthisis are but four annually out of 1000 living—a small proportion, indicating, of course, little tendency to the disease. In the Windward and Leeward Command in the West Indies, the deaths from consumption constitute but one ninth of the total deaths—a low proportion; but the total deaths are 93½ annually out of 1000 living—a high mortality; and the deaths from phthisis are 10½ per 1000—a high mortality from this disease.

Under the fourth class, or diseases of the organs of circulation, 1,596 deaths are recorded, of which 902 occurred in males, and 694 in females, or in the proportion per 1000 of the living of ·262 of the former, and ·195 of the latter. The proportion of these deaths ascribed to heart-disease, Mr. Farr remarks, is much below that stated by Dr. Clendinning, and much below the truth. Our own observation convinces us of the justice of this remark; and we are convinced that many cases in which the primary disease was seated in the heart have been returned under the heads of hydrothorax and dropsy, affections which, in a vast majority of instances, are consecutive.

Five thousand one hundred and fifteen males, and four thousand seven hundred and thirty-five females, died of disease of the digestive organs, and the annual rate of mortality in the males was 1·5, in the females 1·3 out of 1000 living. There is little in the return of individual diseases under this head to call for comment, besides the very remarkable facts that there are but twenty deaths from peritonitis (of which seven were in males and thirteen in females), and *not one from enteritis,* whilst of *gastro-enteritis* there are 3,396! This is "passing strange," but we are by no means convinced that it is "not more strange than true."

"Diseases of the urinary organs destroy five times as many males as females, the rate of mortality of the two sexes, under this head, having been ·199 and ·037 per 1000. This disparity has been ascribed to mechanical causes; but will a mechanical explanation account for the fact, that sixty-eight males and only twenty-seven females died of diabetes? Dr. Yelloly, in a paper published in the Philosophical Transactions, estimated that 1 in 108,000 persons was cut annually for stone in England and Wales. It appears from the table, that 47 in 1,000,000 males, and 5 in 1,000,000 females, die of stone and gravel. The latter, it must be admitted, is a vague term in popular language; but the mortality from stone is certainly 1 in 100,000 annually. Bright's disease is registered "disease of the kidneys." The coagulability of the urine is often undetected by careless practitioners. A female child, aged two years, is stated to have died *suddenly* of diabetes." (p. 105.)

We shall now notice, in a less regular manner, certain sources of mortality. The deaths in childbed amount to 1,265, of which we learn, from an estimate by Dr. Ferguson, seven eighths arise from puerperal fever. The annual rate of mortality by childbirth in females generally, is 3·55 in 10,000; or, calculating only those at a child-bearing age, perhaps 0·8 per 1000. Supposing 290,000 births and miscarriages to have occurred in the period, nearly four in 1000 were fatal to the mothers. It is remarked as a subject of great regret, that 2,500 die in childbirth every year in England and Wales. Under the head of diseases of the skin, we are surprised to observe seven fatal cases of leprosy. Hemorrhage, the effusion of blood, is more fatal in males than females, in the proportion of ·107 to ·060; dropsy, the effusion of serum, more fatal to females than males, in the proportion of ·882 to ·711. Fourteen thousand one hundred and five deaths were ascribed to inflammation of one part or another, consequently one tenth of the fatal cases arose from these affections. Carcinoma was, as might be supposed, more fatal to females than males, the deaths of the former being 875, of the latter 355. Sixty-three deaths were ascribed to starvation, or nearly one annually to a population of 111,000. Mr. Farr's remarks on this point are very just:

"The want of food implies the want of everything else, except water; as firing, clothing, every convenience, every necessary of life, is abandoned at the imperious bidding of hunger. Hunger destroys a much higher proportion than is indicated by the registers in this and every other country; but its effects, like the effects of excess, are generally manifested, indirectly, in the production of diseases of various kinds. The privation is rarely ever absolute; the supply of food is inadequate to supply the wants of the organization, which requires daily animal or vegetable matter containing not less than nine ounces of carbon." (p. 106.)

All this is true, but it is likewise true, as Dr. King has stated, that a *low degree* of vitality arising from deficient nutriment, is not incompatible, in many cases, with a considerable degree of longevity. The stature is stunted, the frame emaciated, and the intellectual and moral qualities are low; and yet these step-children of nature, or rather, we should say, of art, often live long. This, we think, is especially the case where the nutriment has been deficient from the cradle. The organization is then, in many cases, rather defective than vitiated; and though, in frequent instances, the same circumstances produce structural change and disease, yet in others the condition is such as we have described.

The subject of sudden deaths produces some remarks from Mr. Farr worthy of record. He asks, what is the nature of the sudden deaths of which, notwithstanding some difficulties attending the registration of inquests, it appears that ·184 in males and ·118 in females per 1000 are recorded annually? It will be observed, he remarks, that the proportion of sudden deaths is fifty-six per cent. higher in males than in females; the mortality in apoplexy is nineteen per cent., in hemorrhage seventy-eight per cent. higher in males than in females. It is probable, therefore, that sudden death is frequently the effect of hemorrhage. Some strictures are made, with too much truth, on the proceedings of coroners' courts, the rude and cursory examinations after death, of which the results are made the grounds of their decisions; the probability that many cases of poisoning escape detection, from the want of a careful examination of the contents of the stomach; and the uniformity of the verdict of "natural death" on all prisoners who die in gaols, instead of a statement of the cause of death in intelligible language. He says, in concluding this branch of the subject, that "the causes of deaths registered as the result of a solemn, judicial investigation, are the most unintelligible in the register, as it is impossible to attach a specific idea to 'natural death,' to 'visitation of God,' and several other phrases in use in coroners' courts."

His remarks on violent deaths are very important:

"4,845 violent deaths, 3,605 of males, 1,240 of females, were registered; and the annual mortality of males and females under this head was 1,048, and ·348 per 1000, the males having suffered three times as much as females. The excess of males was 2,365, and it more than counterbalanced the mortality of childbirth. If all the violent deaths had been entered in the abstract, the mortality of males under this head would probably have equalled the mortality from typhus. This deserves attention. The individuals carried off by violence, typhus, consumption, and childbirth, are in the meridian of life; and in a political sense their lives are of the highest value. Drowning in rivers and in the sea, burning, injuries in manufactories, explosions in mines, are frequent causes of violent deaths. Suicides are included; the ages and sex of forty-four were distinguished." (p. 107.)

In a note are described several of the causes of violent death. Some of them are sufficiently curious, such as killed by a lion, one male; by the bite of a donkey, one female; by loss of blood from the bite of a ferret, a male aged four months; and by taking *Morrison's pills*, two males. This last is an especially discreditable item. Some of the causes are more unmixedly tragic: thus there were killed by lightning, eleven males, four females (all in the first quarter); by an explosion in a pit in Lamesby district, Chester-le-Street, and the same number in a pit in the Springwell *county* (we believe this to be a misprint for *colliery*), likewise near Chester-le-Street. We regard these two latter items as disgraceful both to the science and humanity of the country. What is the Committee of the House of Commons, which has been sitting for years, on the subject of accidents in coal-mines, doing? We know what it is failing to do.

Mr. Farr's estimates of the comparative mortality in towns and the open country, are among the most important, to the statesman and physician, which the volume contains. The length to which our extracts and remarks have already extended, compel us to dismiss this portion of the subject with more brevity than we wish; but a portion of the more general facts elicited we shall present to the reader.

In table C (p. 148), is an abstract of the deaths from various diseases for the half year in the metropolis, with a population of 1,790,451, on an area of seventy square miles, and in the counties of Cornwall, Dorsetshire, Devonshire, Somersetshire, and Wiltshire, with a population of 1,723,770, on an area of 7,933 square miles. In the metropolis, the total mortality for the period was 24,959; in the counties with the same population, 15,220. Nor is this very great advantage to the country limited to a comparison between it and the metropolis only; for in table D, we have, with results nearly the same, the proportion of deaths from various diseases in the counties of Essex, Gloucester (except Bristol and Clifton), Hereford, Norfolk (except Norwich), Suffolk, Sussex, and Westmoreland; and in the districts of Aston, Bath, Birmingham, Bristol, Cambridge, Carlisle, Clifton, Derby, Dudley, Exeter, Leeds, Leicester, Liverpool, Manchester, Maidstone, Newcastle-on-Tyne, Northampton, Nottingham, Salford, Sheffield, Stoke-on-Trent, Sunderland, Wolverhampton, and West Derby. The cities have a population of 1,762,710 on an area of 677 square miles; the counties one of 1,776,980 on an area of 9,312 square miles. In the former, the mortality in the half-year was 22,994; in the latter, 14,473. It will be observed, that the comparison between the metropolis and other considerable towns is less disadvantageous to the former than might have been expected. The comparison between extensive country districts wide asunder shows a very close correspondence indeed.

In table E, which we regard as one of singular value, we are presented with an abstract of the deaths from twelve classes of fatal diseases in city and country districts, the former with a population of 3,553,161, the latter with a population of 3,500,750. In the cities there perished, from epidemic, endemic, and contagious diseases, 12,766; in the country 6,045; from diseases of the nervous system, the numbers stood respectively 7,705, 3,607; from those of the respiratory organs, 12,619, 7,847; of the organs of circulation, 590, 309; of the digestive organs, 3,467, 1,832; of the urinary organs, 219, 161; of the organs of generation, 460, 265; of the organs of locomotion, 262, 154; of the integumentary system, 62, 55; of uncertain seat, 4,396, 1,657; of age, 2,924, 3,102; of violent deaths, 1,370, 929; of causes not specified, 1,104, 1,657; constituting, collectively, a total of deaths in the cities of 47,953, and in the counties of 29,693.

We subjoin the author's comments on these tables:

"The concentration of the population in cities doubles the deaths from the first two classes of diseases; the ratio of deaths having been as 1 to 2·11, and 1 to 2·13; and upon reference to the individual diseases in tables C, D, it will be observed that the augmentation in the latter class occurs principally in convulsions and hydrocephalus: Deaths by convulsions—counties 1,347, cities 3,723, ratio 1 : 2·76; by hydrocephalus—counties 559, cities 1,540, ratio 1 : 2·75. It has already been intimated, that convulsion is a frequent intercurrent symptom in diarrhœa and diseases of the epidemic class in infants; it may exist, however, as an independent affection, and in that case has clearly, as well as hydrocephalus, with which it is allied, an epidemic character. A similar remark will apply to pneumonia and bronchitis, of which 1,209 cases were registered in the counties, 2,865 in the cities; ratio 1 : 2·37. The pulmonary inflammation was, in many cases, developed in the course of measles, influenza, and other diseases of the first class. The three following diseases, which principally affect adults between the ages of fifteen and sixty-five, show that unhealthy places augment the fatality of diseases in different degrees:

| | Counties. | | Cities. | | Increase per cent. in Cities. |
|---|---|---|---|---|---|
| "Deaths by consumption - - | 5,857 | .... | 8,125 | .... | 39 |
| ,, childbirth - - - | 217 | .... | 372 | .... | 71 |
| ,, typhus - - - - - | 1,564 | .... | 3,456 | .... | 221 |

"This gives the classification a peculiar property. Wherever the absolute mortality is low, the number of deaths in the epidemic class is less than the number in the pulmonary class; and, on the contrary, wherever the deaths in the first class exceed or equal those in the third, it may be affirmed that the absolute mortality is high." (pp. 110-11.)

This last sentence is an explanation of the law of phthisis, which we have already quoted. It seems that it is the variation in the mortality from the epidemic class which produces the contrast between the proportion of deaths from phthisis and the total deaths, in which the law consists.

Mr. Farr, after showing that the Poor Law Enquiry and successive Parliamentary Committees have proved that the excess of the mortality in towns, as compared with the country, does not arise from the difference of the food of the inhabitants of the respective localities, thus expresses himself:

"The source of the higher mortality in cities is, therefore, in the insalubrity of the atmosphere. Every human being expires about 666 cubic feet of gas daily, which, if collected in a reservoir, would destroy other animals; and is constantly producing, in a variety of ways, the decomposition of animal and vegetable matter yielding poisonous emanations in houses, workshops, dirty streets, and bad sewers. The smoke of fires and the products of combustion are also poisonous. All gases and effluvia, like odours, are diffusible; they have a certain force of diffusion, which Professor Graham has expressed numerically; and all the emanations from human habitations in the open country mingle, almost as soon as they escape, in the currents of the atmosphere. But locate, instead of one individual to a square mile of land (the supposed density of population in the uncultivated forests of America, and the stappes of Asia), 200,000 individuals upon a square mile, as soldiers in a camp, and the poison will be concentrated 200,000-fold; intersect the space in every direction by 10,000 high walls, which overhang the narrow streets, shut out the sunlight, and intercept the movements of the atmosphere; let the rejected vegetables, the offal of slaughtered animals, the filth produced in every way, decay in the houses and courts, or stagnate in the wet streets; bury the dead in the midst of the living; and the atmosphere will be an active poison, which will destroy, as it did in London formerly, and as it does in Constantinople now, 5·7 per cent. of the inhabitants annually, and generate, when the temperature is high, recurring plagues, in which a fourth of the entire population will perish. But the health will be little more impaired by residence upon one than 100 square miles, if means can be devised for supplying the 200,000 individuals with 200,000,000 cubic feet of pure air daily, and for removing the principal sources of poisonous exhalations. The latter object is partly accomplished by paved, even streets, by the scavenger, by an abundant supply of water, by large, well-constructed, trapped sewers, and by domestic habits of cleanliness; but it is difficult to perceive how volatile impurities can be removed, and how a stream of uncontaminated air can be supplied where the sun cannot heat the earth and air; where there are no open squares, or the streets are narrow, or the houses are only separated by courts, or built in *cul de sac.*" (pp. 111-12.)

It sounds somewhat paradoxical to say, that *that* civilization which increases the duration of life, should cause the population to be concentrated in large cities—a circumstance which abridges it. Such, however, seems to be the case, and we must therefore conclude, that among many elements of longevity civilization fosters one mighty element of destruction. Still, be it remembered, that Mr. Farr's very valuable facts are deduced from a comparison between rural districts and English towns, the growth of a progressive civilization, and that the older parts of them, now chiefly inhabited by the labouring poor, were formed in the earlier stages of such civilization. We should like much to see a comparison between the mortality of country districts and large towns brought rapidly into existence, under a civilization fully developed—those of the United States for instance.

We are compelled to close here our review of this very important volume. In our last Number we bore testimony, on our own authority, to its very great value, and to the credit justly due to the Registrar-General and Mr. Farr; and we appeal to the extracts in the present one for a confirmation of the truth of this testimony.

